# Ecocriminological analysis of brine in aquatic ecosystems: impacts on *Posidonia oceanica *and the search for restorative justice solutions

**DOI:** 10.12688/openreseurope.16365.1

**Published:** 2023-08-25

**Authors:** Esteban Morelle-Hungría, Pablo Serra

**Affiliations:** 1 Public Law, Universitat Jaume I, Castellón de la Plana, Valencian Community, Spain; 2 Universidad Pontificia Comillas, Madrid, Community of Madrid, Spain

**Keywords:** Green criminology, Posidonia oceanica, desalination, ecological harm, restorative justice.

## Abstract

The consumption of fresh water has generated additional problems in certain territories, along with the consequences of global warming. This has meant that, in the most vulnerable areas, such as the Balearic archipelago in the Mediterranean, alternative water supply systems have been established: desalination plants. However, the ecological impact of these infrastructures is great, mainly affecting aquatic ecosystems. In the light of the above, this paper addresses the ecological harm caused by desalination and brine discharges on a protected and priority species, *Posidonia oceanica*. For this and taking as ethical-legal foundation the theory of ecological justice, a multilevel analysis was carried out from an ecocriminology perspective on the impacts of this authorized practice on *Posidonia* and other species. Finally, a restorative justice view will allow us to understand and envision possible solutions to this ecological harm.

## Introduction

*Posidonia oceanica* (hereafter *Posidonia*) is one of the most important plant species in the ecosystems of the Mediterranean Sea. This importance is mainly because it is an endemic species of this area and due to its role in mitigating the effects of global warming. *Posidonia* meadows are markedly complex ecosystems, in which microhabitats of great species diversity can be found. They are, therefore, a network that enables the ability to feed and respond to the conditions required by different species, taking into account various factors such as light, hydrodynamism and even the needs of predators ( Massutí Pascual *et al.*, 2000). Thanks to these physicochemical and biological characteristics, it is possible to observe the presence of animal species of different characteristics ( Vaquer-Sunyer *et al.*, 2021); *Posidonia* are significant primary producers allowing it to be a source of natural oxygen and, therefore, an important carbon dioxide sink. Likewise, other organoleptic characteristics of this species improve water quality; for example, the foliage of *Posidonia* allows the deposition of suspended particles.

In a territory classified as one of the most vulnerable to the current climatic situation, such as the Balearic Islands, and in one of the areas of greatest risk as is the Mediterranean basin, the preservation of this plant species is essential. Firstly, because of its ecological value, but also for socioeconomic reasons, which enable the presence and development of humans in this territory.

In order to achieve adequate levels of conservation of *Posidonia* in the Balearic Islands, it is necessary to have a comprehensive understanding of the different pressures to which it is subjected, so as to be able to offer holistic political and legal responses. Taking this general objective as a guide, the present research focuses on the ecological harm generated by desalinisation, a human activity that, in turn, is considered necessary to provide certain basic services for our species, such as the supply of drinking water. But before explaining the process of this activity, we must emphasize that the analysis presented here is framed in the field of ecocriminology, that is, the perspective of green criminology that studies ecological harm from an interdisciplinary approach, using methodologies from other sciences such as ecology, and taking as ethical-legal foundation the theory of ecological justice ( Morelle-Hungría, 2020). Thus, it is necessary to carry out an integral analysis of the ecological harm, and, for this, we must carry out a study of the impacts and consequences detected in the different levels of biological organization established in the affected ecosystem. In other words, it is a matter of analyzing the ecological harm generated by this activity through a systems approach.

The practice of desalination can generate an impact that, although authorized by the administration, may alter the aquatic ecosystems. This was the object of study of this work, in which, after explaining the procedure of this activity, we analyzed the impact desalination has on a specific ecosystem, the *Posidonia* meadows, in a territory in which it is of special interest, the Balearic Islands. Subsequently, we analyzed how the consequences generated by this activity can be a challenge that ecocriminology must address, mainly through restorative justice. With this last aspect we want to highlight what science has been indicating to restore the imbalance generated by desalination and thus be able to regenerate or repair the ecological harm produced. In a transversal manner, and as previously mentioned, the discussion and analysis will be guided by those ethical-legal postulates offered by the theory of ecological justice.

In terms of methodology, this study first used a bibliographic search in various scientific databases on the impacts of brine on aquatic ecosystems in the Mediterranean. Once this bibliography was identified, an ecosystem approach was used to carry out a detailed study of the impacts at different scales of the trophic chain existing in the *Posidonia* meadows. Subsequently, based on the proposed classification of the impacts, where the damages have been differentiated into different categories, the possible solutions proposed by the scientific doctrine from the restorative justice have been analyzed. This study is available as a preprint in Zenodo ( Morelle-Hungría, & Serra-Palao, 2023).

## Approach to brine as an environmental problem from the point of view of ecocriminology

The desalination process, or desalination, is a natural process carried out by evaporation. However, humans began to employ this technique for its own benefit ( Ministerio de Sanidad y Política Social, 2008). Currently, this process includes a series of procedures and techniques to which we should briefly refer in order to understand the problems derived from this activity. It is a process by which water is taken from the sea and transformed for human consumption or for use in certain anthropic activities, such as agriculture or other industrial activities. The installation of desalination plants will depend on different decisive factors, especially taking into account the differences in the possible geographical location. This process consists of a series of phases that must be followed until the final result is obtained: the first is the collection of brackish water or seawater; subsequently, the water collected must undergo pre-treatment; finally, in the third phase, the actual treatment takes place. Although it is true that the treatment phase can be carried out using different techniques, the method used by most of the facilities located in our country is by means of the so-called reverse osmosis ( AEDyR, 2019).

Osmosis is a natural biological process that arises spontaneously when two solutions of different concentrations equalize their concentrations to equilibrium, taking place by passing through semi-permeable membranes. These membranes allow the passage of the solution with lower concentration to the higher one, which is possible thanks to the pressure exerted, known as osmotic pressure. This is also a fundamental process in cell biology, so it is necessary to maintain this osmotic equilibrium to ensure the normal biological functioning of the species. It is also possible to carry out the process in reverse, by means of human intervention, applying higher external pressure to the osmotic pressure in such a way that the natural osmotic process is reversed. With this procedure, and by introducing pressure to the solution with a higher concentration of salts, a solution with a lower concentration is obtained when passing through the membranes used. This process produces a stream called brine, known as rejection, which is the water used in osmosis but with a higher salt concentration. The reject water is sent to the sea through outfalls with diffusers, but, previously, most of the facilities located in Spain use mechanisms to minimize environmental impact and low energy consumption ( AEDyR, 2019). The last phase of this desalination process consists of carrying out a post-treatment to adapt the characteristics of the water obtained to its intended use. If the water is to be used for human consumption, it will be necessary to comply with current health and hygiene regulations.

According to current Spanish legislation, there is no national legislation or even regional regulations applicable to brine as a rejection, nor are there any limitations on concentrations or chemical components. In spite of this legislative gap, which we believe should have been filled prior to the construction of this type of industrial plants, projects of a certain size do have to comply with an Environmental Impact Assessment (hereinafter, EIA), in accordance with the provisions of the regulation governing this matter ^1^. This regulation provides that the EIA of the activity must include an analysis and, if applicable, a quantification of its direct or indirect impacts, including the cumulative effects of the project to be authorized. Within this analysis, which is outlined as comprehensive, factors such as biodiversity, the marine environment or climate change must be taken into account, as well as the interaction between all those factors mentioned in art. 45 of Spanish Law 21/2013, on environmental assessment, in the different phases of the project to be approved. It must be taken into account that these evaluations must be carried out prior to the installation of the activity, having to verify compliance with those conditions arising from environmental regulations.

Likewise, in order to carry out the emission of the discharge of rejection to the authorized areas, generally located in coastal areas, it is necessary to have an authorization to discharge the brine by the competent administrative authority for the regulation of marine pollution. According to AEDyR (2019),

“... Desalination plant operation projects must include an exhaustive environmental monitoring program with the aim of implementing a series of guidelines to protect possible sensitive areas from excess salinity generated by brine discharges. The main objective of these programs is to be able to scientifically analyze the behavior of the discharge during the operation phase of the desalination plant and to reduce any type of impact associated with it.” ( AEDyR, 2019).

The coastal areas where the outfalls of these facilities are located usually have a great biodiversity, which must be controlled in accordance with current regulations. This monitoring program should cover not only the characteristics of the water where the brine discharge will be deposited (controlling pH, turbidity or the concentration of some pollutants such as nitrates, among others), but also analyses should be carried out at different levels to verify the alteration of the ecosystem as a whole.

**Figure 1.**
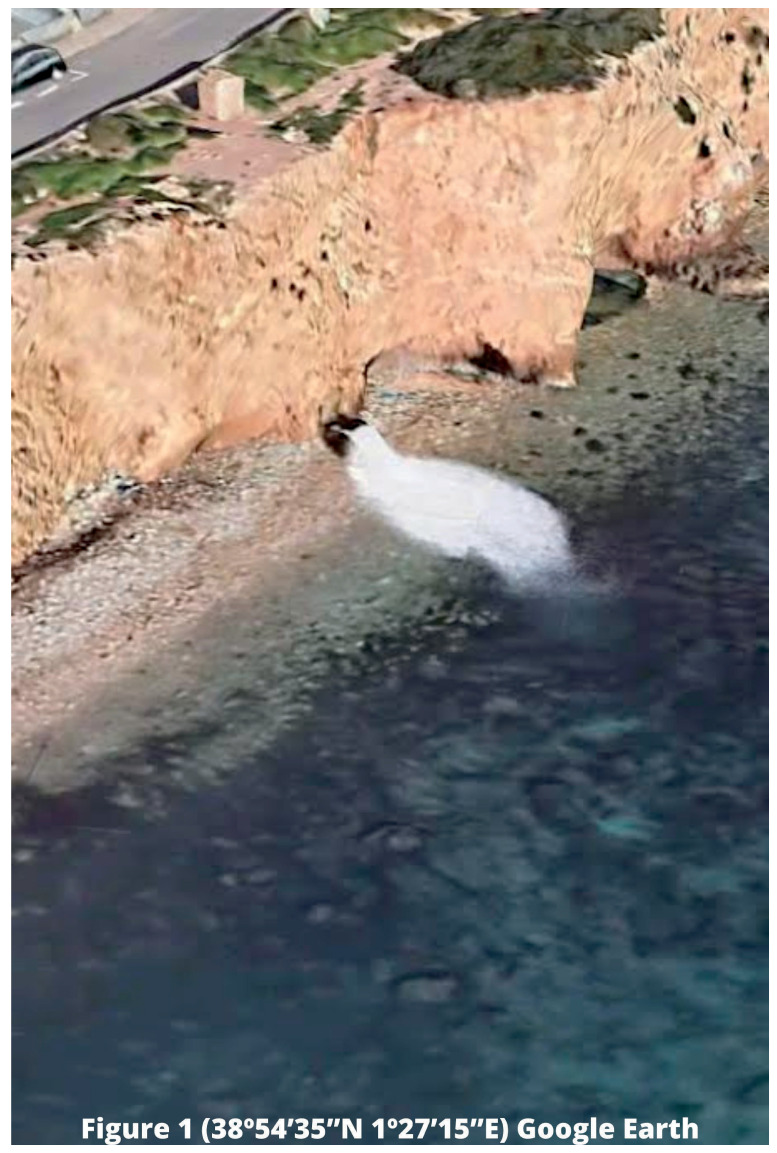
Location of outfall in Talamanca Bay, Ibiza (Balearic Islands). Source: Google Earth 2023.

As we have indicated, the installation of outfalls responsible for discharging brine into the marine environment is usually located in coastal areas (see Figure 1), where there may be numerous habitats or ecosystems of interest. In fact, and entering already in the analyzed case, the brine discharged by the desalination plant located in the city of Ibiza, Balearic Islands, has been considered by different territorial media as a "poison for Posidonia" ( Gaceta Náutica, 2016). *Posidonia* meadows are included as a habitat of community interest, an area of special conservation, defined in Annex I of Directive 92/43/EEC, known as the Habitats Directive ^2^, where its great biological diversity and the conditions of the species have derived that this superior plant is, in addition, a protected species at national and regional level.

In relation to the impacts of brine on marine phanerogams, and especially on *Posidonia,* the Spanish Ministry of Health and Social Policy established the following in 2008:

“... Regarding the impact of brine discharges on submerged biological communities, it is well known that Posidonia oceanica has a low tolerance to slight increases in salinity. According to the studies carried out to date, and in accordance with the precautionary principle, increases in salinity from 39.1 PSU ^3^ produce a reduction in growth, the appearance of necrosis in the tissues and premature leaf fall. On the other hand, other species characteristic of these environments (e.g., sea urchins and mysidaceans) may also be affected” ( Ministerio de Sanidad y Política Social, 2008, p. 183).

Brine has physical and chemical properties that are different from the environment in which it is dumped. For this reason, it is advisable to rigorously analyze the impacts that may be produced on this environment. The EIAs carried out should also consider the cumulative effect at three different levels: short, medium and long term. The high concentrations of salts in this discharge can reach 69 g/l ( Gaceta Náutica, 2016), being considered hypersalinity as it is twice higher than what we can naturally find in seawater. In addition, we can also find along with this high concentration of mineral salts some chemical elements that are used for desalination processes, such as sodium chloride, magnesium chloride or potassium sulfate, among others. On the other hand, it can be observed how the temperature also differs, reaching almost five degrees of difference to that of the medium where it is poured ( Gaceta Náutica, 2016).

**Figure 2.**
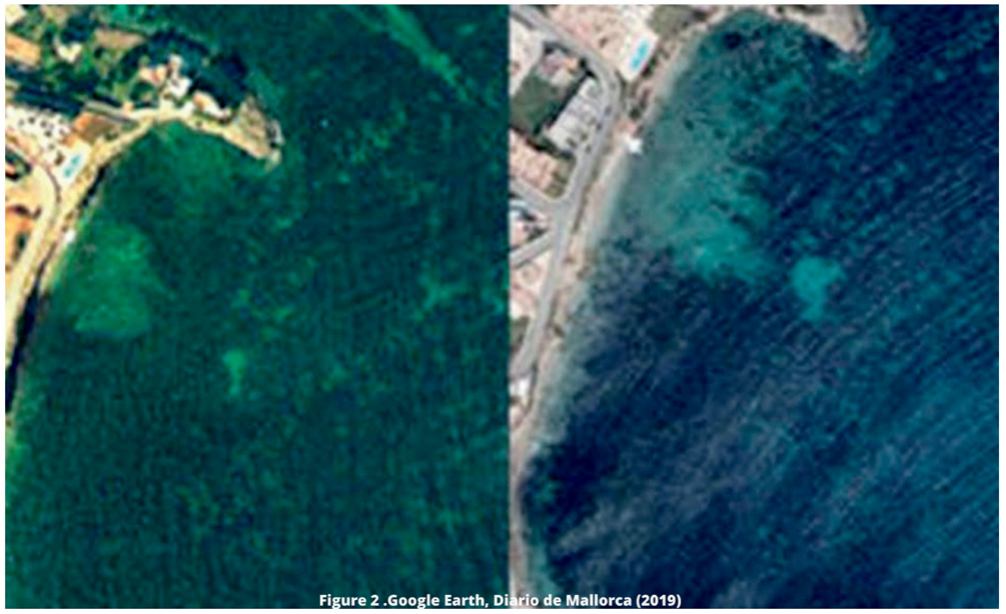
Retreat of *Posidonia* meadows in Talamanca Bay in two different periods. Source: Diario de Mallorca, 2019. Images obtained through Google Earth. https://mas.diariodemallorca.es/inevitable-efecto-desaladoras-sobre-posidonia-baleares/

In the Balearic Islands there are up to eight desalination plants, controlled by the Regional Government's Department of the Environment. All those in operation use reverse osmosis. As mentioned in previous paragraphs, we are going to focus on the desalination plant in the city of Ibiza, which dates back to 1994. The own administrations of the island of Ibiza ( Diez-Caballero *et al.*, 2018), have highlighted the detected retreat of the *Posidonia* meadows in the vicinity of the outlet of the outfall located in Talamanca Bay in the town of Ibiza. A significant retreat has been evidenced in terms of the extent of this priority habitat in the area where the brine is discharged, which can be observed through images obtained with Google Earth ( Figure 2) and published in the Diario de Mallorca (2019).

**Figure 3.**
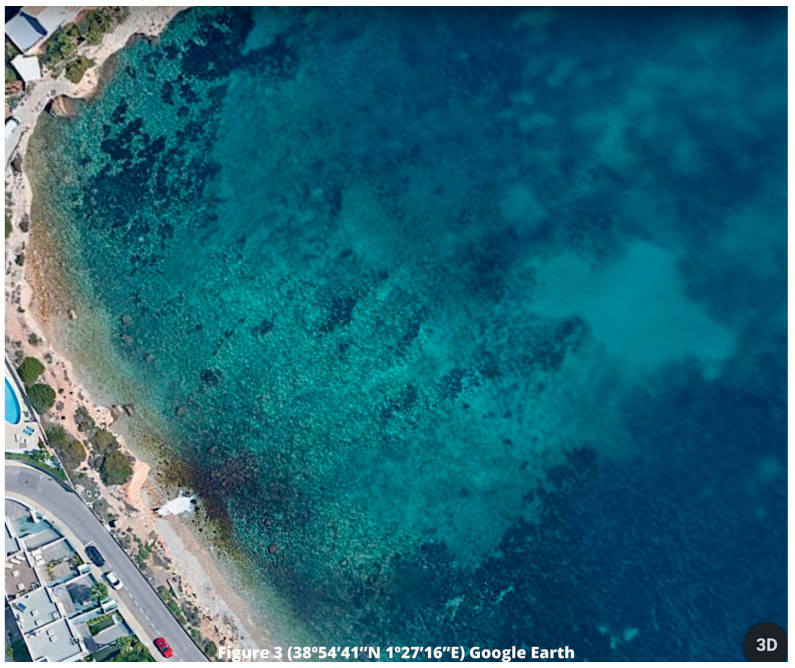
Affected area in Talamanca Bay, Ibiza, Balearic Islands. Source: Google Earth, 2023.

Currently, we can observe that the retreat of *Posidonia* meadows in the affected area has worsened ( Figure 3). Although it is true that we cannot establish a single direct relationship with this activity, as it coexists with different anthropic pressures. Despite this, we maintain that these discharges are one of the current problems facing this species, which is reflected in the next section. In this sense, we must take into account that since 1994 the area of Talamanca Bay receives this dumping, observing a clear retreat in the *Posidonia* meadows, which is evidenced by those 70 meters, approximately, of water clarity ( Costa, 2018; Diario de Mallorca, 2019; Roig, 2015).

As some studies indicate ( Sobrado *et al.*, 2022), this area is in a situation of degradation derived from the anthropic pressures that are detected on it, where we must take into account both the discharge of sewage water ( Sobrado *et al.*, 2018), as well as the dumping of brine, which together significantly affects this protected species of special importance for its ecosystem services. Such is the level of pressure exerted in the area that up to 40% of the *Posidonia* in Talamanca Bay is dead ( Periódico de Ibiza, 2023).

## Brine pressure on *Posidonia oceanica* in the Balearic archipelago: analysis of impacts from an ecosystem perspective

This section emphasizes the different impacts caused by brine, both on the *Posidonia* meadow and on other species. The ecocriminological analysis of the ecological harm caused by the brine spill requires a brief preliminary note on the ecosystemic composition of this habitat, which is configured as one of the areas of greatest biodiversity on the Mediterranean coast, given the plurality of animal and plant species that can be found there. Thus, some studies highlight the importance of its primary production, constituting the basis for more than 1,500 species of great trophic diversity ( Massutí Pascual *et al.*, 2000). On the other hand, authors such as Vaquer-Sunyer *et al.* (2021, p.3) have pointed out the existence in this habitat of two well differentiated natural spaces for those species that cannot move, called sessile: the foliar stratum, for species with photoaffinity; and the basal part, where species that seek environments without light are found. Likewise, these same authors highlight the presence in the *Posidonia* meadow of numerous animal species that can move, such as molluscs, crustaceans, echinoderms and fish.

### Impacts on the habitat

As has been indicated, there is a great variety of species directly linked to *Posidonia*, as it is a source of primary production for them. The different scientific studies analyzed in the framework of this work and described below, show the causal relationship between the alteration of the biological conditions of the benthic communities and the dumping of brine. Recently, Blanco-Murillo *et al.* (2022) analyzed the regression patterns of *Posidonia* from 1984 to 2014 in the coastal zone of the city of Alicante (Spain), focusing on the possible causal relationships between the management of the protection of these ecosystems and the impacts detected by anthropogenic activities. The great anthropic pressure exerted in this area has affected numerous species, but especially the *Posidonia* meadow in the area *:* activities such as waste dumping, maritime traffic, trawling, industrial activities and, specifically, brine discharges from desalination plants, have led to the destruction of this priority and protected habitat (2022, p. 5). As these authors warn, during the years under study there has been a regression of *Posidonia* meadows of almost 25%, having gone from 2,500 hectares in 1984 to 1,881 in 2014, with an average loss of approximately 21 hectares per year during the aforementioned period (ibid, p. 3). In other Mediterranean territories whose drinking water supply depends to a large extent on desalination, such as the island of Cyprus, the relationship between the discharge of brine derived from the desalination process and the regression of *Posidonia* meadows has also been clearly detected, and the carbon dioxide emissions associated with this activity have also been highlighted ( Xevgenos *et al.*, 2021).

Brine discharge represents a real threat to benthic communities in general, mainly due to the physicochemical characteristics of this discharge. As an illustration, we can mention the work of Cambridge *et al.* (2019), where the effects of brine on a species of *Posidonia*, *Posidonia australis,* are exposed, having detected an increase in the speed and symptoms of stress in adult plants.

Regarding salinity levels and the specific effects on *Posidonia* derived from an increase in these levels, the study conducted by Sánchez-Lizaso *et al.* (2008) showed significant damage to the growth and length of leaves, the appearance of necrotic tissue on them and, in addition, a higher mortality rate. According to this research, salinities higher than 39.1 practical salinity units (PSU) cause significant negative impacts and, in fact, when reaching 45 PSU, half of the plants in the study died in just 15 days. Tests were even carried out on the impacts caused by these changes in salinity on species connected to *Posidonia*, being detected at salinity above 41 PSU mortality in some animal species, such as *Paracentrotus lividus* (sea urchin) or *Leptomysis posidoniae* (mysidacean), in conjunction with other factors such as increased temperature ( Sánchez-Lizaso *et al.*, 2008, p. 604). Likewise, on the island of Formentera (Balearic Islands), within the framework of this research, salinities between 38.4 and 39.8 PSU were detected in the area closest to the outfall, with the identification of substances such as nitrates and phosphates in concentrations higher than those of the receiving waters. In this area it was observed that the *Posidonia* meadows affected showed a reduction in the length of leaves and a physiological affectation of the plants, which according to these authors could indicate that eutrophication was the cause of the degradation in the area of the outfall, but, at a different distance from it, the salinity recorded was slightly lower (37.8 and 39.3 PSU) and the species no longer showed these signs of eutrophication. In any case, signs of salinity stress were observed, such as necrosis marks on leaves and a lower presence of animal species in the meadows analyzed ( Sánchez-Lizaso *et al.*, 2008, p. 605).

Along the same lines, the study carried out by Gacia *et al.* (2007) on the island of Formentera and with data from 2001, shows a great sensitivity of *Posidonia* to brine discharges from desalination plants, a sensitivity that is explained by the hypersaline conditions and, in addition, by eutrophication, an indirect impact that would be associated with brine discharges ( Gacia *et al.*, 2007, p. 589). Although it is true that this study does not reach a robust conclusion on the establishment of a specific threshold limit for salinity, it does propose a value of 39.3 PSU ( Gacia *et al.*, 2007, p. 589), which would be in line with the results obtained by other studies ( Fernández-Torquemada & Sánchez-Lizaso, 2005). However, other authors recommend a lower threshold, namely 38.5 PSU, for any discharge with hypersalinity that could affect *Posidonia oceanica* meadows in the western Mediterranean (in this sense, *vid.* Navarro-Barrio *et al.*, 2021, pp. 2 and 5; Sánchez-Lizaso *et al.*, 2008, p. 606).

In addition, there are multiple studies that focus on the relationship between increased salinity and certain effects on *Posidonia*, such as a reduction in the number of germinated seeds or delayed germination periods, as indicated by Fernández-Torquemada and Sánchez-Lizaso (2013) citing other authors ( Caye *et al.*, 1992; Conacher *et al.*, 1994; Harrison, 1991; Hootsmans *et al.*, 1987; Loques *et al.*, 1990; Phillips *et al.*, 1983). Hypersalinity can also affect leaf growth according to other research ( Balestri & Cinelli, 2003; Balestri *et al.*, 2009; Belzunce *et al.*, 2008), in which a lower number of leaves and a modification in leaf length are observed in the presence of salinity higher than 40 PSU ( Gacia *et al.*, 2007). Thus, we can demonstrate the causal relationship between an increase in hypersalinity and the affectation of the territorial expansion and extension of this species. Despite this, the limitations established by Gacia *et al.*, (2007) suggest that studies should continue in conjunction with other parameters such as temperature or pH, characteristics that could also affect the distribution and extension of this plant species. On the other hand, some studies, such as Malfeito *et al.*, (2005), show the importance of diluting the brine discharge to minimize the impact on possible affected ecosystems. Salinity in the discharge area, according to this study, averaged 39.5 PSU. However, it is worth mentioning that in this research, focused on the Xàbia desalination plant (province of Alicante, Spain), the discharge area is the Fontana Canal. This is a small area, capable of assuming a greater volume of discharge, with a better gradient, less biological diversity and far from the *Posidonia* meadows (p. 91). The area affected by the brine, therefore, has not resulted in significant impacts on this species (pp. 93–94).

The different scientific evidence reviewed in this section highlights the importance of a risk analysis where tolerance thresholds are established for *Posidonia oceanica* meadows ( Navarro-Barrio *et al.*, 2021), as this is the species that is mainly affected by brine spills. However, it should be taken into account that actions to protect *Posidonia* against this type of pressure should be articulated prior to the start of the activity; in this way, with the integral analysis within the EIAs, effective mechanisms could be introduced. As some authors indicate, if the measures are adopted once the *Posidonia species has* regressed, it will not be able to recover effectively ( González-Correa *et al.*, 2005).

### Impacts on other species

As mentioned in previous paragraphs, the great biodiversity existing in *Posidonia* meadows may mean that this habitat is not the only one affected by brine spills. The change in salinity conditions generated by the difference in concentration of mineral salts can modify the characteristics needed for the survival of different animal species ( Frank *et al.*, 2019). More recent studies ( Bianchelli *et al.*, 2022) indicate that there is a link between hypersalinity and the effect on certain animal species, specifically crustaceans, polychaetes and other species of meiofauna (small sessile organisms, invertebrates), reaching more than 90% of species of this taxon in the areas near the outfall of the discharge.

Templado (2014) indicates that brine discharges from desalination plants require a cumulative effect with other pressures so that these damages can be of such intensity that it would be difficult to recover the affected area. Thus, the capacity for adaptation would be limited in view of the different anthropic impacts that are usually detected in areas such as Talamanca Bay. Some of the species found in this habitat, which may be affected by the regression and even disappearance of this source of primary production of vital importance to the marine ecosystem, are listed below. It is worth mentioning that more than a thousand species have been identified as being linked to these endemic habitats. First of all, and merely as an example, we will refer to those that feed on this superior plant, which would be especially linked to the changes in the *Posidonia* meadows. We have the salpa fish ( *Sarpa salpa*), the green turtle ( *Chelonia mydas*) and some sea urchins, such as the *Paracentrotus lividus*. There are several organisms that remain attached to the rhizomes of this plant and even on the leaves, such as the hydrozoan *Aglaophenia harpago*, the bryozoan *Lichenopora radiata* and *Electra posidoniae*, the latter species forming white strips on the leaves that can be seen. There are also some species of crustaceans, such as *Idotea hectica* or a nudibranch like *Diaphorodoris papillata*. Other invertebrates of interest are those known as starfish, *Asterina pancerii* ( Arenas-Camps, 2016). One of the most characteristic animals of the *Posidonia oceanica* meadows is the nacre ( *Pinna nobilis*), the largest mollusk in the Mediterranean, which can reach a meter in length and lives with part of its body buried in the sand. Finally, some fish, such as *Serranus scriba*, known as cow and the sucker fish, *Opeatogenys gracilis* ( Arenas-Camps, 2016).

All these species linked to *Posidonia* may suffer an indirect impact as a result of the regression or disappearance of the species itself, as it is their habitat and, in part, their main substrate for primary production. Thus, taking into account the set of pressures that can be generated and that have been analyzed, we can establish that there may be an indirect link between the situation of the plant species and the rest of the species that live and inhabit it. This question is of vital importance, taking into account that one of the main sources of income in the area analyzed is tourism and the activities that can be carried out in marine and coastal areas, which depend essentially on the quality of the water.

## Proposals from restorative justice

At this point, we can consider the pressure of brine on *Posidonia* meadows as a real ecological harm, having materialized in all those multidimensional impacts addressed in the previous section. Considering the negative effects observed both on *Posidonia* and on a great diversity of species dependent on it, the point of view offered by restorative justice can make a positive contribution to the way we understand this damage and to the design of possible responses to it ( Varona, 2021, p. 42). In any case, and as we already mentioned in the introduction of this paper, the possible solutions that could emerge from restorative justice must be understood within the framework of ecological justice. This theory takes as its starting point the interrelation and interdependence between the different living organisms and the non-living natural environment that make up an ecosystem, recognizing the intrinsic value of the ecosystem as a whole ^4^ to support its protection and thus maintain its ecological integrity, which would refer to the "combination of biodiversity and ecosystemic processes that characterize an ecosystem at a given time, so that the goods and services provided by that ecosystem are available continuously over time" ( Serra-Palao, 2020, p. 244). If ecological justice incorporates the totality of organisms into the justice community itself ( Baxter, 2005), these ecosystem goods and services must be considered from a non-anthropocentric perspective, and, therefore, the needs of other organisms to flourish and develop adequately must be taken into account. This means, in short, that human activity must respect certain ecological limits. Based on the above, in cases of ecological harm, restorative justice should be aimed at recovering those ecosystemic conditions that have been altered by the anthropic activity causing the damage, which are necessary for the affected species to flourish and develop adequately. Thus, the proposed perspective provides a socioecological effort incorporating empirical analysis, which can improve the management and governance of natural resources ( Tepper, 2023, p. 130).

This intimate connection between green criminology, ecological justice and restorative justice has already been highlighted by numerous authors, who have even formulated concepts such as “green restorative justice” ( Varona, 2020; Varona, 2021) or “environmental restorative justice” ( Forsyth *et al.*, 2021). However, we propose here the concept of ‘ecological restorative justice’, as we believe it would be more in line with the perspective and methodology of ecocriminology, as ecological restorative justice would also transfer methodologies from ecology and establish a relationship of dependence with the scientific conclusions coming from it, which would facilitate the configuration of restorative processes in which the biological and ecological needs of the species involved would be adequately identified.

One aspect that reinforces the importance of this analysis of ecological harm is the use of ecocriminological terminology in the scientific study, since, as Bearzi (2020) points out, it could give the impression that the regulatory measures incorporated are carried out in search of true ecological justice, prioritizing, moreover, that those responsible for ecological harm must answer for their actions while at the same time incorporating reparation mechanisms ( Ruggiero & South, 2010; Ruggiero & South, 2013). Thus, the consequences of desalination coupled with other pressures on these ecosystems result in the acceleration of a possible collapse of these ecosystems ( Pauly, 2019), which could be qualified as an ecological crime from ecocriminology.

In this case, the linkage of the ecological harm generated by this activity has been established, so that from ecological restorative justice we can propose a double response: on the one hand, measures of prevention and mitigation; on the other hand, of repair and adaptation. But before presenting these measures, we must insist on the importance of having the participation of the different actors involved and especially of the Administration, the main guarantor of the good condition of ecosystems. In this sense, a comprehensive mechanism must be formulated in order to reach the greatest possible consensus on the means to be applied ( Meyer, 2015, p. 171), through, for example, a working group specifically dedicated to this ecological harm and composed of the actors involved, in which the needs of the non-human species affected would be represented through experts from various scientific fields.

As for prevention and mitigation measures in the Talamanca Bay area, a scientifically agreed maximum salinity threshold should be established and an adequate periodicity of observations should be established. It would also be desirable to analyze the possible use of brine for another purpose, for example, ‘brine mining’, which allows the extraction of the elements present in this type of discharge. Elements such as phosphorus, rubidium, magnesium, indium, cesium, potassium chloride, and sodium chloride can be extracted, being economically viable as they can be used for the production of gypsum or calcium carbonate, among others. However, this type of technology has not been incorporated in most facilities ( Morillo *et al.*, 2014).

Secondly, it is necessary to establish instruments for the repair and eventual adaptation of the affected environment to the new situation. As the most recent studies suggest, the repopulation of species affected by anthropogenic action offers a potential that is being untapped to address major global challenges, such as climate change ( Schmitz *et al.*, 2023).

One of the techniques used for the restoration and regeneration of this plant has been the transplanting of seedlings from seeds ( Balestri *et al.*, 1998), making this technique one of the best options compared to other more traditional ones such as the regeneration technique using adult rhizomes. This is a technique already known and studied with positive results as it allows improvements such as attachment to the sediment and allows the seed to root more effectively ( Celdrán-Sabater, 2011). This is a challenge for both scientists and administrations; however, the restoration potential has been evidenced by science itself ( Duarte *et al.*, 2020; McAfee *et al.*, 2021). Despite these studies, these lines of research should continue to be expanded, especially in areas with hypersalinity, as it has also been found that there is a direct relationship between high salinities and the evolution of restoration ( Escandell-Wescott, 2022).

## Conclusion

From our ecocentric vision and in the search for true ecological justice, there are many parameters to consider in order to establish a relationship of lesser impact between humans and marine ecosystems. These parameters not only refer to more specific issues related to certain impacts, but also to reflect on the most essential, the abuse of a resource such as seawater, the abstraction of water from the Mediterranean, when this activity is even controversial on the part of the administrations of the island of Ibiza, especially when scientific data show a negative causal relationship between the dumping of brine with the regression of *Posidonia*. This is an activity protected and authorized by the competent authorities but which, in turn, generates a direct and indirect impact on the ecosystem itself and endangers ecosystem services. We could consider that these activities are legal but generate illegitimate ecological harm to aquatic ecosystems and therefore to nature itself ( South, 2014, p. 379).

We believe that criminology should continue to analyze the ecological harm caused by anthropic activities within the broader framework of ecological justice. Picking up the witness of Lynch, Stretesky and Long (2015) a global challenge that the human species itself has generated has been raised, so it is necessary that, from our discipline, we contribute to the study of ecological justice from criminological research attending to the fact that our species is part of the affected ecosystems and, mainly, being the cause of the problem that endangers different species and ecosystems.

One of the measures that should be adopted, in the case analyzed, would be to improve the EIAs. To this end, scientific advances should be taken into account, for example, including a long-term comprehensive multilevel study, so as to prioritize those measures that achieve a lower negative impact on the ecosystem ( Panagopoulos, 2022).

The importance of collaborative and planned governance will be essential to achieve significant sustainable improvement. Environmental assessment processes should include alternative analyses of the analyzed desalination systems, the environmental impacts of desalination activities on marine ecosystems can be minimized. In addition, seagrass monitoring mechanisms can be incorporated with a monitoring system so that negative impacts on seagrass meadows can be detected in time and acted upon with real data ( Sola *et al.*, 2020, p. 8).

## Data Availability

No data associated with this article.
