# Ecocriminological analysis of brine in aquatic ecosystems: impacts on *Posidonia oceanica *and the search for restorative justice solutions

**DOI:** 10.12688/openreseurope.16365.2

**Published:** 2024-02-16

**Authors:** Esteban Morelle-Hungría, Pablo Serra-Palao

**Affiliations:** 1 Public Law, Universitat Jaume I, Castellón de la Plana, Valencian Community, Spain; 2 Universidad Pontificia Comillas, Madrid, Community of Madrid, Spain

**Keywords:** Green criminology, Posidonia oceanica, desalination, ecological harm, restorative justice.

## Abstract

The consumption of fresh water has generated additional problems in certain territories, along with the consequences of global warming. This has meant that in the most vulnerable areas, such as the Balearic archipelago in the Mediterranean, alternative water supply systems have been established: desalination plants. However, the ecological impact of these infrastructures is great, mainly affecting aquatic ecosystems. In light of the above, this paper addresses the ecological harm caused by desalination and brine discharges on a protected and priority species, *P. Oceanica*. Taking as ethico-legal foundation the theory of ecological justice, a multilevel analysis was carried out from an ecocriminology perspective on the impacts of this authorized practice on *P. Oceanica* and other species. Finally, a restorative justice view will allow us to understand and envision possible solutions to this ecological harm.

## Introduction

*Posidonia oceanica* (hereafter *P. oceanica*) is one of the most important plant species in the ecosystems of the Mediterranean Sea. This importance is mainly because it is an endemic species of this area and due to its role in mitigating the effects of global warming. *P. oceanica* meadows are markedly complex ecosystems in which microhabitats of great species diversity exist. They are, therefore, a network that enables the ability to feed and respond to the conditions required by different species, taking into account various factors such as light, hydrodynamism and even the needs of predators ( Massutí Pascual *et al.*, 2000). Thanks to these physicochemical and biological characteristics, it is possible to observe the presence of animal species of different characteristics ( Vaquer-Sunyer *et al.*, 2021); *P. oceanica* is a significant primary producer, a source of natural oxygen and, therefore, an important carbon dioxide sink. Likewise, other organoleptic characteristics of this species improve water quality; for example, the foliage of *P. oceanica* allows the deposition of suspended particles.

In a territory classified as one of the most vulnerable to the current climatic situation, such as the Balearic Islands (Spain), and in one of the areas of greatest risk, as it is the Mediterranean basin, the preservation of this plant species is essential. Firstly, because of its ecological value, but also for socioeconomic reasons, which enable the presence and development of humans in this territory.

In order to achieve adequate levels of conservation of *P. oceanica* in the Balearic Islands, it is necessary to have a comprehensive understanding of the different pressures on this species so as to be able to offer holistic political and legal responses. This general objective guides the present research, which focuses on the ecological harm generated by desalination, a human activity that, in turn, is considered necessary to provide certain services considered basic for our species, such as the supply of drinking water. But before explaining the process of this activity, we must emphasize that the analysis presented here is framed in the field of ecocriminology, that is, the perspective of green criminology that studies ecological harm from an interdisciplinary approach, using methodologies from other sciences such as ecology, and taking as ethico-legal foundation the theory of ecological justice ( Morelle-Hungría, 2020). Thus, it is necessary to carry out an integral analysis of the ecological harm. For this, we must carry out a study of the impacts and consequences detected in the different levels of biological organization established in the affected ecosystem. In other words, it is a matter of analyzing the ecological harm generated by this activity through a systems-based approach ( Tourangeau, 2022).

The practice of desalination can generate an impact that, although authorized by the administration, alters the aquatic ecosystems. This was the object of study of this work, in which, after explaining the procedure of this activity, we analyzed the impact desalination has on a specific ecosystem, the *P. oceanica* meadows, in a territory in which it is of special interest, the Balearic Islands (Spain). Subsequently, we analyzed how the consequences generated by this activity can be a challenge that ecocriminology must address, mainly through restorative justice. With this last aspect, we want to highlight what science has been indicating to restore the imbalance generated by desalination and thus be able to regenerate or repair the ecological harm produced. In a transversal manner, the discussion and analysis will be guided by the ethico-legal theory of ecological justice.

In terms of methodology, this study first used a literature search in various scientific databases on the impacts of brine on aquatic ecosystems in the Mediterranean. After identifying the scientific literature, we used an ecosystem approach to carry out a detailed study of the impacts at different scales of the trophic chain in the *P. oceanica* meadows. Subsequently, based on the proposed classification of the impacts, where the damages have been differentiated into different categories, the possible solutions proposed by the scientific doctrine have been analyzed from the restorative justice approach. This study is available as a preprint in Zenodo ( Morelle-Hungría, & Serra-Palao, 2023).

## Approach to brine as an environmental problem from the point of view of ecocriminology

Desalination is a natural process carried out by evaporation. However, humans began to employ this technique for their benefit ( Ministerio de Sanidad y Política Social, 2008). Currently, this process includes a series of procedures and techniques to which we should briefly refer in order to understand the problems derived from this activity. It is a process by which water is taken from the sea and transformed for human consumption or use in certain anthropic activities, such as agriculture or other industrial activities. The installation of desalination plants will depend on different decisive factors, especially taking into account the differences in the possible geographical location. This process consists of a series of phases before obtaining the final result: the first is the collection of brackish water or seawater; subsequently, the water collected must undergo pre-treatment; finally, in the third phase, the actual treatment takes place. Although the treatment phase can be carried out using different techniques, the method used by most of the facilities located in Spain is the so-called reverse osmosis ( AEDyR, 2019).

Osmosis is a natural biological process that arises spontaneously when two water solutions of different concentrations equalize their concentrations to equilibrium, taking place by passing through semi-permeable membranes. These membranes allow the passage of the solution with a lower concentration to the higher one, which is possible thanks to the pressure exerted, known as osmotic pressure. This process is also fundamental in cell biology, so it is necessary to maintain this osmotic equilibrium to ensure the normal biological functioning of the species. It is also possible to reverse the process by means of human intervention, applying higher external pressure to the osmotic pressure in such a way that the natural osmotic process is reversed. With this procedure, and by introducing pressure to the solution with a higher concentration of salts, a solution with a lower concentration is obtained when passing through the membranes used. This process produces a stream called brine, known as rejection, which is the water used in osmosis but with a higher salt concentration. The rejected water is sent to the sea through outfalls with diffusers. Most of the facilities located in Spain use mechanisms to minimize the environmental impact and low energy consumption ( AEDyR, 2019). The last phase of this desalination process consists of conducting a post-treatment to adapt the characteristics of the obtained water to its intended use, and this is done on the permeate from the reverse osmosis process. If the water is for human consumption, it will be necessary to comply with current health and hygiene regulations.

According to current Spanish legislation, there is no national legislation or even regional regulations applicable to brine as a rejection, nor are there any limitations on concentrations or chemical components. In spite of this legislative gap, which we believe should have been considered prior to the construction of these industrial plants, projects of a certain size do have to submit an Environmental Impact Assessment (hereinafter, EIA) in accordance with the regulations governing this matter ^1^. This regulation provides that the EIA of the activity must include an analysis and, if applicable, a quantification of its direct or indirect impacts, including the cumulative effects of the project under the process of authorization. Within this analysis, factors such as biodiversity, the marine environment or climate change must be taken into account, as well as the interaction between all those factors mentioned in art. 45 of Spanish Law 21/2013, in the different phases of the project. These evaluations must be carried out prior to the installation of the activity, having to verify compliance with those conditions arising from environmental regulations.

Likewise, in order to carry out the emission of the discharge of rejection to the authorized areas, generally located in coastal areas, it is necessary to have an authorization to discharge the brine by the competent administrative authority for the regulation of marine pollution. According to AEDyR (2019),

“... Desalination plant operation projects must include an exhaustive environmental monitoring program with the aim of implementing a series of guidelines to protect possible sensitive areas from excess salinity generated by brine discharges. The main objective of these programs is to be able to scientifically analyze the behavior of the discharge during the operation phase of the desalination plant and to reduce any type of impact associated with it.” ( AEDyR, 2019).

The outfalls of these facilities are generally located in coastal areas, where there may exist a large number of biological diversities. Thus, it must be subject to supervision in accordance with current regulations. This monitoring program should cover not only the characteristics of the water where the brine discharge will be deposited (controlling pH, turbidity, or the concentration of some pollutants such as nitrates, among others), but also analyses should be carried out at different levels to verify the alteration of the ecosystem as a whole.

**Figure 1.**
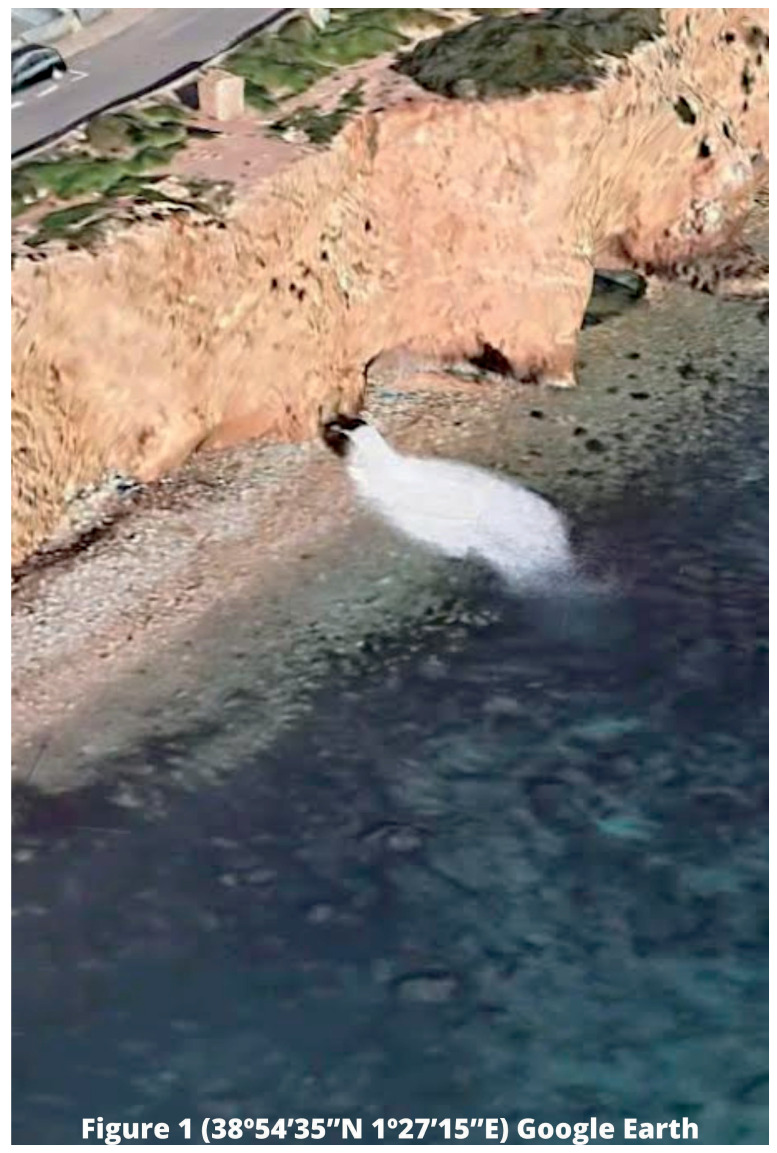
Location of outfall in Talamanca Bay, Ibiza (Balearic Islands). Source: Google Earth 2023.

Figure 1 illustrates the installation of outfalls responsible for discharging brine into the marine environment in coastal areas, where there may be numerous habitats or ecosystems of interest. It is important to note that *P. oceanica* meadows are listed as a habitat of community interest, an area of special conservation, defined in Annex I of Directive 92/43/EEC, known as the Habitats Directive ^2^, where its great biological diversity and the conditions of the species have derived that this plant is, in addition, a protected species at the national and EU level.

In relation to the impacts of brine on marine phanerogams, and especially on *P. oceanica,* the Spanish Ministry of Health and Social Policy established the following in 2008:

“... Regarding the impact of brine discharges on submerged biological communities, it is well known that Posidonia oceanica has a low tolerance to slight increases in salinity. According to the studies carried out to date, and in accordance with the precautionary principle, increases in salinity from 39.1 PSU ^3^ produce a reduction in growth, the appearance of necrosis in the tissues and premature leaf fall. On the other hand, other species characteristic of these environments (e.g., sea urchins and mysidaceans) may also be affected” ( Ministerio de Sanidad y Política Social, 2008, p. 183).

Brine has physical and chemical properties that are different from the environment in which it is dumped. For this reason, it is advisable to analyze the impacts that may be produced rigorously. The EIAs carried out should also consider the cumulative effect in the short, medium and long term. The high concentrations of salts in this discharge can reach 69 PSU ( Gaceta Náutica, 2016), which is considered hypersalinity as it is almost twice higher than what we can naturally find in seawater. Along with this high concentration of mineral salts, we can also find some chemical elements used for desalination processes. On the other hand, the temperature also differs, reaching almost five degrees of difference from that of the medium where it is poured ( Gaceta Náutica, 2016). However, brine can present salinities of 60-70 PSU, but that does not mean that these salinities are detectable in the marine environment. Natural dispersal usually reduces salinity to 40-45 PSU in the first meters ( Fernández-Torquemada *et al.*, 2009)

**Figure 2.**
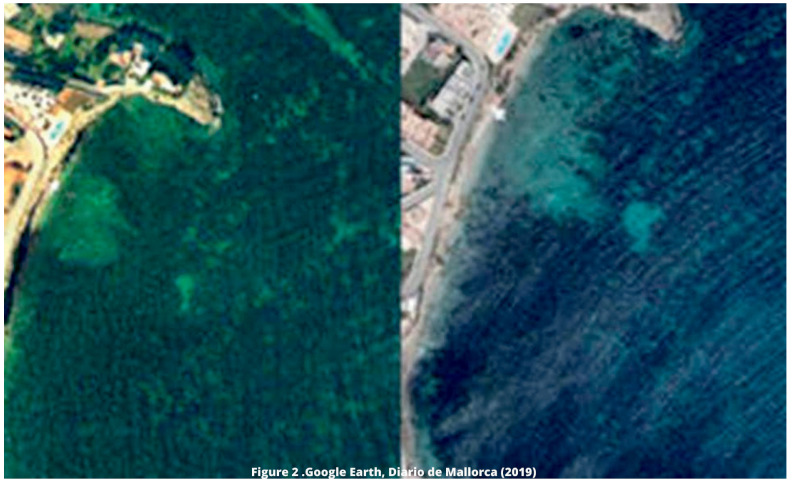
Retreat of *Posidonia* meadows in Talamanca Bay in two different periods. Source: Diario de Mallorca, 2019. Images obtained through Google Earth. https://mas.diariodemallorca.es/inevitable-efecto-desaladoras-sobre-posidonia-baleares/

In the Balearic Islands, there are up to eight desalination plants under the control of the Regional Government's Department of the Environment. All those in operation use reverse osmosis. As mentioned in previous paragraphs, we are going to focus on the desalination plant in the city of Ibiza, which dates back to 1994. The administrations of the island of Ibiza ( Diez-Caballero *et al.*, 2018), have highlighted the detected retreat of the *P. oceanica* meadows in the vicinity of the outlet of the outfall located in Talamanca Bay in the town of Ibiza. A significant retreat has been evidenced in terms of the extent of this priority habitat in the area where the brine is discharged, which can be observed through images obtained with Google Earth ( Figure 2) and published in the Diario de Mallorca (2019).

**Figure 3.**
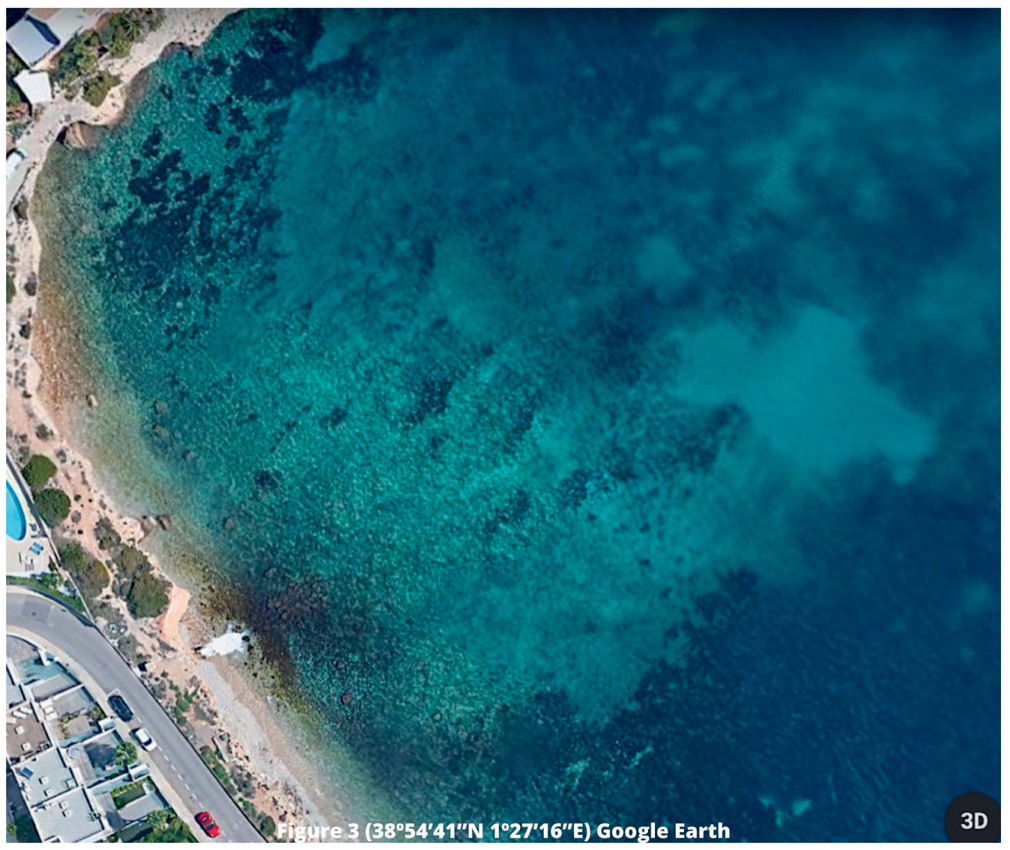
Affected area in Talamanca Bay, Ibiza, Balearic Islands. Source: Google Earth, 2023.

Currently, we can observe that the retreat of *P. oceanica* meadows in the affected area has worsened ( Figure 3). However, we cannot establish a single direct relationship with this activity, as it coexists with different anthropic pressures. Despite this, we maintain that these discharges are one of the current problems facing this species, which is reflected in the next section. In this sense, we must take into account that since 1994, the area of Bahía de Talamanca has received this dumping, observing a clear retreat in the *P. oceanica* meadows, which is evidenced by those 70 meters, approximately, of water clarity ( Costa, 2018; Diario de Mallorca, 2019; Roig, 2015).

As some studies indicate ( Sobrado *et al.*, 2022), this area is in a situation of degradation derived from the anthropic pressures that are detected on it, where we must take into account both the discharge of sewage wáter ( Sobrado *et al.*, 2018), as well as the dumping of brine, which together significantly affects this protected species of special importance for its ecosystem services. Such is the level of pressure exerted in the area that up to 40% of the *P. oceanica* in Talamanca Bay is dead ( Periódico de Ibiza, 2023).

## Brine pressure on Posidonia oceanica in the Balearic archipelago: analysis of impacts from an ecosystem perspective

The present section emphasizes the different impacts caused by brine, both on the *P. oceanica* meadow and on other species. The ecocriminological analysis of the ecological harm caused by the brine spill requires a brief preliminary note on the ecosystemic composition of this habitat, which is configured as one of the areas of greatest biodiversity on the Mediterranean coast, given the plurality of animal and plant species that can be found there. Thus, some studies highlight the importance of its primary production, constituting the basis for more than 1,500 species of great trophic diversity ( Massutí Pascual *et al.*, 2000). On the other hand, authors such as Vaquer-Sunyer *et al.* (2021, p.3) have pointed out the existence in this habitat of two well-differentiated natural spaces for those species that cannot move, called sessile: the foliar stratum, for species with photoaffinity; and the basal part, for species that seek environments without light. Likewise, these same authors highlight the presence in the *P. oceanica* meadow of numerous animal species that can move, such as molluscs, crustaceans, echinoderms and fish.

### Impacts on the habitat

There are also animal species within this habitat that consume primary production of *P. oceanica*. Different scientific studies show the causal relationship between the alteration of the biological conditions of the benthic communities and the dumping of brine. Recently, Blanco-Murillo *et al.* (2022) analyzed the regression patterns of *P. oceanica* from 1984 to 2014 in the coastal zone of the city of Alicante (Spain), focusing on the possible causal relationships between the management of the protection of these ecosystems and the impacts detected by anthropogenic activities. The great anthropic pressure exerted in this area has affected numerous species, but especially the *P. oceanica* meadow in the area: activities such as waste dumping, maritime traffic, trawling, industrial activities and, specifically, brine discharges from desalination plants, have led to the destruction of this priority and protected habitat ( 2022, p. 5). During the years under study, these authors warn that there has been a regression of *P. oceanica* meadows of almost 25%, having gone from 2,500 hectares in 1984 to 1,881 in 2014, with an average loss of approximately 21 hectares per year during this period (ibid, p. 3). In other Mediterranean territories whose drinking water supply depends to a large extent on desalination, such as the island of Cyprus, the relationship between the discharge of brine derived from the desalination process and the regression of *P. oceanica* meadows has also been clearly detected. Other studies have pointed out the carbon dioxide emissions associated with this activity ( Xevgenos *et al.*, 2021).

Brine discharge represents a real threat to benthic communities in general, mainly due to the physicochemical characteristics of this discharge. As an illustration, we can mention the work of Cambridge *et al.* (2019), where the effects of brine on *P. oceanica australis* are exposed, detecting an increase in the speed and symptoms of stress in adult plants and highlighting the detrimental role brine might have on *P. oceanica* beyond hypersalinity itself, in connection with the conclusions obtained in Blanco-Murillo *et al.* (2023).

Regarding salinity levels and the specific effects on *P. oceanica* derived from an increase in these levels, the study conducted by Sánchez-Lizaso *et al.* (2008) showed significant damage to the growth and length of leaves, the appearance of necrotic tissue on them and, in addition, a higher mortality rate. According to this research, salinities higher than 39.1 PSU cause significant negative impacts and, in fact, when reaching 45 PSU, half of the plants in the study died in just 15 days. Tests were also conducted on the impacts caused by these changes in salinity on species connected to *P. oceanica*, detecting mortality in some animal species at salinity above 41 PSU, such as *Paracentrotus lividus* (sea urchin) or *Leptomysis posidoniae* (mysidacean), in conjunction with other factors such as increased temperature ( Sánchez-Lizaso *et al.*, 2008, p. 604). Likewise, on the island of Formentera (Balearic Islands), within the framework of this research, salinities between 38.4 and 39.8 PSU were detected in the area closest to the outfall, with the identification of substances such as nitrates and phosphates in concentrations higher than those of the receiving waters. In this area, the affected *P. oceanica* meadows showed a reduction in the length of the leaves and a physiological affectation of the plants, which, according to these authors, could indicate that eutrophication was the cause of the degradation in the area of the outfall; but, at a different distance from it, the salinity recorded was slightly lower (37.8 and 39.3 PSU), and the species no longer showed these signs of eutrophication. In any case, signs of salinity stress were observed, such as necrosis marks on leaves and a lower presence of animal species in the analyzed meadows ( Sánchez-Lizaso *et al.*, 2008, p. 605).

Along the same lines, the study carried out by Gacia *et al.* (2007) on the island of Formentera and with data from 2001, shows a great sensitivity of *P. oceanica* to brine discharges from desalination plants. This sensitivity is explained by the hypersaline conditions and, in addition, by eutrophication, an indirect impact that would be associated with brine discharges ( Gacia *et al.*, 2007, p. 589). Although, indeed, this study does not reach a robust conclusion on the establishment of a specific threshold limit for salinity, it does propose a value of 39.3 PSU ( Gacia *et al.*, 2007, p. 589), which would be in line with the results obtained by other studies ( Fernández-Torquemada & Sánchez-Lizaso, 2005). However, other authors recommend a lower threshold, namely 38.5 PSU, for any discharge with hypersalinity that could affect *P. oceanica* meadows in the western Mediterranean (in this sense, *vid.* Navarro-Barrio *et al.*, 2021, pp. 2 and 5; Sánchez-Lizaso *et al.*, 2008, p. 606).

In addition, multiple studies focus on the relationship between increased salinity and certain effects on *P. oceanica*, such as a reduction in the number of germinated seeds or delayed germination periods, as indicated by Fernández-Torquemada and Sánchez-Lizaso (2013) citing other authors ( Caye *et al.*, 1992; Conacher *et al.*, 1994; Harrison, 1991; Hootsmans *et al.*, 1987; Loques *et al.*, 1990; Phillips *et al.*, 1983). Hypersalinity can also affect leaf growth according to other research ( Balestri & Cinelli, 2003; Balestri *et al.*, 2009; Belzunce *et al.*, 2008), in which a lower number of leaves and a modification in leaf length are observed in the presence of salinity higher than 40 PSU ( Gacia *et al.*, 2007). Thus, we can demonstrate the causal relationship between an increase in hypersalinity and the affectation of the territorial expansion and extension of this species. Despite this, the limitations established by Gacia *et al.* (2007) suggest that studies should continue in conjunction with other parameters, such as temperature or pH, characteristics that could also affect the distribution and extension of this plant species. On the other hand, some studies, such as Malfeito *et al.* (2005), show the importance of diluting the brine discharge to minimize the impact on possible affected ecosystems. Salinity in the discharge area, according to this study, averaged 39.5 PSU. However, it is worth mentioning that in this research, focused on the Xàbia desalination plant (province of Alicante, Spain), the discharge area is the Fontana Canal. This is a small area, capable of assuming a greater volume of discharge, with a better gradient, less biological diversity and, most importantly, far from the *P. oceanica* meadows (p. 91). The area affected by the brine, therefore, did not result in significant impacts on this plant species (pp. 93–94).

The different scientific evidence reviewed in this section highlights the importance of a risk analysis where tolerance thresholds are established for *P. oceanica* meadows ( Navarro-Barrio *et al.*, 2021), as this is the species that is mainly affected by brine spills. However, actions to protect *P. oceanica* against this type of pressure should be articulated prior to the start of the activity. Thus, together with the integral analysis of EIAs, effective mechanisms could be introduced. As some authors indicate, if the measures are adopted once the *P. oceanica species* has regressed, it will not be able to recover effectively ( González-Correa *et al.*, 2005).

However, it is crucial to bear in mind that the main cause of degradation is not a single, isolated anthropogenic impact. The accumulation of these impacts on seagrass meadows in heavily populated, touristic areas causes significant detrimental effects on these fragile underwater ecosystems. *P. oceanica* faces threats from various human activities associated with coastal tourism, with anchoring and mooring being one of the main sources of disturbance ( Morelle-Hungria, 2018).

For example, the frequent use of recreational boats poses the risk of directly damaging seagrass meadows by uprooting plants and disturbing the marine substrate. In addition, the accumulation of waste, sewage discharges, and chemical pollution from tourist activities are major threats. These elements compromise water quality and negatively affect plant health, weakening their ability to perform key ecological functions ( Blanco-Murillo *et al.*, 2023). Recreational underwater activities, such as widespread diving, can also cause direct physical damage to seagrass meadows and disturb surrounding marine habitats. This direct impact adds to the loss of habitat and biodiversity, affecting numerous marine species that depend on seagrass for feeding and reproduction. The resulting loss of biodiversity can disrupt the marine ecosystem and affect resilience to environmental change.

Finally, the cumulative effect of these anthropogenic impacts can alter coastal dynamics, increasing vulnerability to erosion and reducing the ecosystem's capacity to act as a carbon sink. Addressing these challenges requires responsible management and conservation measures that balance the needs of tourism with the long-term preservation of these crucial marine ecosystems.

### Impacts on other species

As mentioned in previous paragraphs, the great biodiversity existing in *P. oceanica* meadows may mean that this habitat is not the only one affected by brine spills. The change in salinity conditions generated by the difference in concentration of mineral salts can modify the survival characteristics of animal species ( Frank *et al.*, 2019). More recent studies ( Bianchelli *et al.*, 2022) indicate that there is a link between hypersalinity and the effect on certain animal species, specifically crustaceans, polychaetes and other species of meiofauna (small sessile organisms, invertebrates), reaching more than 90% of species of this taxon in the areas near the outfall of the discharge.

Templado (2014) indicates that brine discharges from desalination plants require a cumulative effect with other pressures so that these damages can be of such intensity that it would be difficult to recover the affected area. Thus, the capacity for adaptation would be limited in view of the different anthropic impacts that are usually detected in areas such as Talamanca Bay. Some of the species found in this habitat, which may be affected by the regression and even disappearance of this source of primary production of vital importance to the marine ecosystem, are listed below. It is worth mentioning that more than a thousand species have been identified as being linked to these endemic habitats. First of all, and merely as an example, we will refer to those that feed on this superior plant, which would be especially dependent on the changes in the *P. oceanica* meadows. We have the salpa fish ( *Sarpa salpa*), the green turtle ( *Chelonia mydas*) and some sea urchins, such as the *Paracentrotus lividus*. Several organisms remain attached to the rhizomes of this plant and even on the leaves, such as the hydrozoan *Aglaophenia harpago*, the bryozoan *Lichenopora radiata* and *Electra posidoniae*, the latter species forming visible white strips on the leaves. There are also some species of crustaceans, such as *Idotea hectica* or a nudibranch like *Diaphorodoris papillata*. Other invertebrates of interest are those known as starfish, *Asterina pancerii* ( Arenas-Camps, 2016). One of the most characteristic animals of the *P. oceanica* meadows is the nacre ( *Pinna nobilis*), the largest mollusk in the Mediterranean. It can reach a meter in length and lives with part of its body buried in the sand. Finally, some fish, such as *Serranus scriba*, known as cow and the sucker fish, *Opeatogenys gracilis* ( Arenas-Camps, 2016).

All these species linked to *P. oceanica* may suffer an indirect impact as a result of the regression or disappearance of the species itself, as it is their habitat and, in part, their main substrate for primary production. Thus, taking into account the set of analyzed pressures, we can establish that there may be an indirect link between the situation of the plant species and the rest of the species that live and inhabit *P. oceanica* meadows. This question is of vital importance, as some of the main sources of income in the area are tourism and those activities performed in marine and coastal areas, which depend essentially on the quality of the water.

### Proposals from restorative justice

At this point, we can consider the pressure of brine on *P. oceanica* meadows as ecological harm, having materialized in all those multidimensional impacts addressed in the previous section. Considering the negative effects observed both on *P. oceanica* and on a great diversity of species dependent on it, the point of view offered by restorative justice can make a positive contribution to the way we understand this damage and to the design of possible responses to it ( Varona, 2021, p. 42). In any case, and as we already mentioned in the introduction of this paper, the possible solutions that could emerge from restorative justice must be understood within the ethico-legal framework of ecological justice. This theory takes as its starting point the interrelation and interdependence between the different living organisms and the non-living natural environment that make up an ecosystem, recognizing the intrinsic value of the ecosystem as a whole ^4^ to support its protection and thus maintain its ecological integrity. The notion of ecological integrity refers to the "combination of biodiversity and ecosystemic processes that characterize an ecosystem at a given time, so that the goods and services provided by that ecosystem are available continuously over time" ( Serra-Palao, 2020, p. 244). If ecological justice incorporates the totality of organisms into the justice community itself ( Baxter, 2005), a non-anthropocentric perspective is essential in protecting the ecological integrity of a specific ecosystem. Therefore, the ecological needs of other organisms to flourish and develop adequately must be taken into account. In other words, human activity must respect certain ecological limits. Based on the above, in cases of ecological harm, restorative justice should be aimed at recovering those anthropogenically altered ecological conditions necessary for the flourishing of the affected species. Thus, the proposed perspective —restorative justice within a broader ethico-legal framework of ecological justice— provides a socioecological effort incorporating empirical analysis, which can improve the management and governance of ecosystems ( Tepper, 2023, p. 130).

This intimate connection between green criminology, ecological justice and restorative justice has already been highlighted by numerous authors, who have even formulated concepts such as ‘green restorative justice’ ( Varona, 2020; Varona, 2021) or “environmental restorative justice” ( Forsyth *et al.*, 2021). However, we propose the concept of ‘ecological restorative justice’, as we believe it would be more in line with the perspective and methodology of ecocriminology, as ecological restorative justice would also transfer methodologies from ecology and establish a strong relationship with the scientific conclusions coming from this field, which would facilitate the configuration of restorative processes in which the biological and ecological needs of the species involved would be adequately identified. The incorporation of restorative measures against activities that may cause environmental harms to this plant should be a priority. In this sense, it would be interesting to replace financial fines with repopulation projects in the affected areas.

As Bearzi (2020) points out, it could give the impression that the regulatory measures incorporated are carried out in search of true ecological justice, prioritizing, moreover, that those responsible for ecological harm must answer for their actions while at the same time incorporating reparation mechanisms ( Ruggiero & South, 2010; Ruggiero & South, 2013). However, to achieve this, comprehensive measures must be put in place. The consequences of desalination, coupled with other pressures on these ecosystems, result in the acceleration of a possible collapse of these ecosystems ( Pauly, 2019), which could qualify as an ecological crime from ecocriminology.

From an ecological restorative justice approach, we can propose a double response to the present case: on the one hand, measures of prevention and mitigation; on the other hand, of repair and adaptation. But before presenting these measures, we must insist on the relevance of guaranteeing the participation of all different actors involved, not only the public administration and private companies but, most importantly, citizens. In this sense, a comprehensive mechanism must be formulated in order to reach the greatest possible consensus on which means should be applied ( Meyer, 2015, p. 171). For example, a working group consisting of different stakeholders could be established to address this ecological harm. By adopting an ecological restorative justice approach, the needs of affected non-human species could be represented through experts from various scientific fields.

As for prevention and mitigation measures in the Talamanca Bay area, three measures are worth highlighting. First, a scientifically agreed maximum salinity threshold with adequate periodicity of observations. Second, it would also be desirable to analyze the possible use of brine for another purpose, for example, ‘brine mining’, which allows the extraction of the elements present in this type of discharge. Elements such as phosphorus, rubidium, magnesium, indium, and caesium can be extracted, being economically viable as they can be used for the production of gypsum or calcium carbonate, among others. However, this type of technology has not been incorporated in most facilities ( Morillo *et al.*, 2014). Third, another mitigation measure should be the adaptation of diffusion mechanisms in the outfalls to dilute the concentration of salts that are discharged into the sea.

Furthermore, it is necessary to establish instruments for the repair and eventual adaptation of the affected environment. As the most recent studies suggest, the repopulation of species affected by anthropogenic action offers a potential that is unexplored in addressing major global challenges, such as climate change ( Schmitz *et al.*, 2023).

One technique used for restoring and regenerating this plant is transplanting seedlings ( Balestri *et al.*, 1998). This technique is one of the best options compared to other more traditional ones such as using adult rhizomes. The technique of transplanting seedlings has had positive results as it allows improvements such as attachment to the sediment and allows the seed to root more effectively ( Celdrán-Sabater, 2011). It is evident that this is a challenge for both scientists and administrations; however, the restoration potential of this technique has been evidenced by science itself ( Duarte *et al.*, 2020; McAfee *et al.*, 2021). Despite these studies, these lines of research should continue to be expanded, especially in areas with hypersalinity, as it has also been found that there is a direct relationship between high salinities and the evolution of restoration ( Escandell-Wescott, 2022).

## Conclusion

In light of compelling scientific literature and evidence establishing a direct causal relationship between brine impact and its deleterious effects on this habitat, as well as other resident species, it is important to remain prudent. While increased resources are crucial for minimizing the impacts of this activity, it is imperative to recognize that the cumulative effect of various pressures poses the greatest negative impact on *P. oceanica*.

Effective mitigation, adaptation, and restoration mechanisms are essential to address the multifaceted challenges faced by *P. oceanica*. Beyond the specific concern on brine impact, a holistic strategy is necessary to confront the broader array of stressors contributing to the degradation of this marine habitat. Proactive measures are essential not only for preserving this plant species but also for strengthening the resilience of the entire ecosystem against cumulative anthropogenic pressures.

In this context, strategic planning and interdisciplinary collaboration are paramount. Implementing measures that encompass mitigating immediate threats, adapting practices to sustainable alternatives, and actively engaging in habitat restoration efforts are pivotal steps toward ensuring the long-term health and viability of *P. oceanica* and its associated ecosystems. The complexity of these challenges underscores the need for a comprehensive and concerted effort to safeguard the delicate balance of these marine habitats.

An ecological restorative justice approach requires considering multiple parameters to establish a harmonious relationship between humans and marine ecosystems. These parameters not only refer to more specific issues related to certain impacts but also reflect on the most essential, i.e., extracting water from the Mediterranean, when this activity is even controversial on the part of the public administrations of the island of Ibiza. Addressing this situation becomes even more urgent when scientific data has shown a negative causal relationship between the dumping of brine and the regression of *P. oceanica*. Brine discharge is protected and authorized by the competent authorities. However, it also generates a direct and indirect impact on the ecosystem itself and endangers ecosystem services. We could consider these activities to be legal but generate illegitimate ecological harm to aquatic ecosystems and, therefore, to nature itself ( South, 2014, p. 379).

Ecocriminology should continue to analyze the ecological harm caused by anthropic activities within the broader framework of ecological justice. As Lynch, Stretesky and Long (2015) noted, humanity has created a significant global challenge, and our discipline must contribute to the study of ecological justice through criminological research. We must acknowledge that our species is not only affected by these environmental issues but is also the primary cause of them, endangering various ecosystems and species.

In the case under consideration, it is advisable to improve the EIAs. This can be achieved by incorporating the latest scientific research, such as conducting a thorough, multi-level study over the long term. In doing so, priority should be given to measures that have a minimal negative impact on the ecosystem, as recommended by Panagopoulos (2022).

The importance of collaborative and planned governance will be essential to achieve significant sustainable improvement. Environmental assessment processes should include alternative analyses of the desalination systems, focusing on minimizing the environmental impacts of desalination activities on marine ecosystems. It is worth noting that the incorporation of seagrass monitoring mechanisms can play a crucial role in the timely detection of negative impacts on seagrass meadows. These monitoring systems could ensure that decision-makers have access to accurate and reliable data, which could help in taking appropriate action to mitigate the adverse effects on seagrass Meadows ( Sola *et al.*, 2020, p. 8).

With regard to collaborative governance in response to a marine spill impacting seagrass meadows, an ecological restorative justice approach would involve several key steps. First, identifying affected parties, including local communities, fishers, and environmental organizations. Facilitating an inclusive dialogue between these parties, the responsible actor, and environmental experts is crucial to understanding the full extent of the damage. Encouraging the responsible actor to take accountability and acknowledge the impact promotes transparency. Coordinating efforts for restoration, such as cleaning the affected seagrass and implementing preventative measures against future spills, is a collaborative process. Seeking appropriate compensation mechanisms for affected parties, including ecosystem rehabilitation measures, is integral to the restorative approach. Additionally, promoting educational programs and awareness initiatives emphasizes the importance of marine conservation and helps prevent future incidents. Establishing a long-term monitoring system ensures the ongoing effectiveness of restoration measures and continued compliance with responsibilities. Restorative approaches aim not only to punish offenders but also to restore affected relationships and rehabilitate the impacted environment through collective efforts and active participation from all parties involved.

The implementation and use of desalination plants pose a significant ethical dilemma, weighing their ecological impact against the pressing need for access to drinking water. Ethically addressing this dilemma requires a comprehensive assessment of the environmental impact associated with desalination. This comprehensive assessment involves considering factors such as energy consumption, the residual brine discharged in the process, and potential adverse effects on surrounding marine ecosystems. In pursuit of an ethical solution, prioritizing the development of more efficient and sustainable desalination technologies is crucial. Continuous research and the implementation of technological advancements can contribute to minimizing the environmental footprint of these facilities holistically. In this sense, exploring and promoting the use of renewable energy sources to power desalination plants can reduce their carbon footprint.

However, it is important to mention that desalination should not be regarded as the sole solution. It is crucial to adopt an integrated water management approach, including conservation, reuse, and recycling strategies. This broader approach helps reduce exclusive reliance on desalination and addresses water resource sustainability more holistically. Community involvement is also paramount in decision-making related to desalination. Engaging the local communities promotes an adequate consideration of their needs and concerns, strengthening the moral legitimacy of the decisions made. Additionally, ensuring equitable access to drinking water is imperative, addressing considerations of social and economic justice.

Ultimately, the ethical dilemma between desalination and environmental impact is best resolved through a balanced and sustainable approach. This involves a continued commitment to research and development, community engagement, and consideration of comprehensive alternatives to ensure not only immediate access to drinking water but also the long-term preservation of our environment.

## Data Availability

No data associated with this article.

## Footnotes

^1^ In accordance with Spanish Law 21/2013, of December 9, 2013, on environmental assessment (BOE no. 296, of 11/12/2013), water desalination or desalobration facilities with a new or additional volume greater than 3,000 cubic meters per day must undergo simplified EIA. In this regard, *vid.* art. 7.2.a), arts. 45 to 48 and Annex II of the aforementioned law.

^2^ Council Directive 92/43/EEC of 21 May 1992 on the conservation of natural habitats and of wild fauna and flora (OJ L 206, 22.7.1992). http://data.europa.eu/eli/dir/1992/43/oj

^3^ Practical Salinity Units. This is a unitless variable adopted in the late 1970s by the Joint Oceanographic Standards Panel which recommended its definition in this unit. It is the ratio of seawater conductivity to a standard KCl (Potassium Chloride) solution. An older salinity scale is ppt (10–3), where salinity is defined by "the salt content is the weight of inorganic salts contained in 1 kg of seawater if all the bromide and iodide are replaced by an equivalent amount of oxides" ( Knudsen, 1901).

^4^ We understand that an ecosystem has intrinsic value because it is valuable in itself considered or as an end in itself, independently of any external valuation or whether it is valuable as a means to other ends.
