# Supplementary material for: Ecocriminological analysis of brine in aquatic ecosystems: impacts on Posidonia oceanica and the search for restorative justice solutions
Source: Open Res Eur. 2024 Feb 16;3:130. Originally published 2023 Aug 25. [Version 2] doi: 10.12688/openreseurope.16365.2 (PMC10873548; doi:10.12688/openreseurope.16365.2)
Supplement: Peer review history

# Reviewer report: Reviewer response for version 2

**DOI:** 10.21956/openreseurope.18652.r38259

**Published:** 2024-04-12

**Authors:** Fabio Blanco-Murillo

**Affiliations:** 1 University of Alicante, Alicante, Spain

I find the corrections and amendments made by the authors to improve the current manuscript and make it more suitable for indexing.

Is the argument persuasive and supported by appropriate evidence?

Partly

Is the work clearly and cogently presented?

Partly

Is the topic of the essay discussed accurately in the context of the current literature?

Partly

Does the essay contribute to the cultural, historical, social understanding of the field?

Yes

Reviewer Expertise:

Environmental impact of desalination projects and ecotoxicology

I confirm that I have read this submission and believe that I have an appropriate level of expertise to confirm that it is of an acceptable scientific standard.

# Reviewer report: Reviewer response for version 2

**DOI:** 10.21956/openreseurope.18652.r38256

**Published:** 2024-03-12

**Authors:** Jordi Pagès

**Affiliations:** 1 Centre d'Estudis Avançats de Blanes, Girona, Spain

I have now read the manuscript and I’d like to approve it.

Is the argument persuasive and supported by appropriate evidence?

Partly

Is the work clearly and cogently presented?

Partly

Is the topic of the essay discussed accurately in the context of the current literature?

Partly

Does the essay contribute to the cultural, historical, social understanding of the field?

Yes

Reviewer Expertise:

Ecology

I confirm that I have read this submission and believe that I have an appropriate level of expertise to confirm that it is of an acceptable scientific standard.

# Reviewer report: Reviewer response for version 2

**DOI:** 10.21956/openreseurope.18652.r38257

**Published:** 2024-02-23

**Authors:** Gema Varona

**Affiliations:** 1 University of Basque Country, Leioa, Spain

I have no further comments to make.

Is the argument persuasive and supported by appropriate evidence?

Partly

Is the work clearly and cogently presented?

Yes

Is the topic of the essay discussed accurately in the context of the current literature?

Partly

Does the essay contribute to the cultural, historical, social understanding of the field?

Yes

Reviewer Expertise:

Criminology, Victimology, Restorative Justice

I confirm that I have read this submission and believe that I have an appropriate level of expertise to confirm that it is of an acceptable scientific standard.

# Reviewer report: Reviewer response for version 1

**DOI:** 10.21956/openreseurope.17668.r36653

**Published:** 2024-02-16

**Authors:** Teresa Fajardo del Castillo

**Affiliations:** 1 University of Granada, Granada, Spain

Marine pollution from different sources - ship source or land source - is already one of the main concerns associated with the triple planetary crisis: climate change, biodiversity loss and pollution. 50 years after the Declaration on the Human Environment that was adopted in the first major United Nations conference for the protection of the environment held in Stockholm in 1972, we have to remember that it affirmed the obligation of states to take all possible measures to prevent pollution of the seas. However, States have carried out an exploitation of the seas which, far from preserving them, has multiplied the sources and risks of marine pollution. The study carried out by Esteban Morelle-Hungría and Pablo Serra-Palao addresses the origin of the problem of a type of marine pollution that has a particular impact on the Mediterranean sea and its possible solution. The dumping of brine from seawater desalination processes is analysed with the ecocentric approach of green criminology, and with the richness provided by a multidisciplinary approach that includes a rigorous review of the scientific studies on the subject as well as the necessary references to the regulatory framework and its environmental, criminological, economic and social implications. As this study shows, this activity is among those authorised by the administrations but without them sufficiently fulfilling their obligations to review and update permits and authorisations in the light of scientific knowledge, the requirements of the ecosystem approach and the data warning of the serious damage caused to *Posidonia meadows*, one of the marine flora species crucial to the preservation of an ecosystem as vulnerable as that of the Balearic Islands. Focusing their analysis on the *Posidonia meadows* on the island of Ibiza, which are classified as a habitat of community interest and special area of conservation, under the Habitats Directive, the authors provide the solutions that ecocriminology can address, mainly through restorative justice.

 One of the most relevant contributions of this work is to have identified one of the regulatory gaps that require an urgent response from the European and Spanish authorities in the face of the lack of provision in the authorisations for the construction of desalination plants, of measures on the adequate and must needed treatment of brine waste. The authors point out that "in spite of this legislative gap, which we believe should have been filled prior to the construction of this type of industrial plant, projects of a certain size do have to comply with an Environmental Impact Assessment, in accordance with the provisions of the regulation governing this matter. (...) It must be taken into account that these evaluations must be carried out prior to the installation of the activity, having to verify compliance with those conditions arising from environmental regulations” (page no. 4). Thus, authors consider that programs of control and monitoring should be carried out at different levels to verify the alteration of the ecosystem as a whole, since the *Posidonia meadows* have been affected greatly by human activities. After an exhaustive review of scientific studies on the effects of different sources of pollution, such as waste dumping, maritime traffic, trawling, industrial activities and, in particular, the dumping of brine from desalination plants, the authors warn that these activities have led to a regression of *Posidonia meadows*. Therefore, among its proposals, they call for responses to this type of activity to be preventive.  As in so many other cases where highly vulnerable marine ecosystems and species are affected, if action is taken only in reaction to serious damage, they will not be able to recover effectively. For this reason, they urge for the introduction of effective mechanisms as part of the integrated analysis within the EIAs, to be articulated prior to the start of the activity. The proposed management model is also exportable to other countries in the Mediterranean basin where this problem is becoming more and more serious, and to other parts of the world. See the growing problem of brine discharges from desalination in the Persian Gulf (1)

 Then, the authors focus on restorative justice solutions that should be understood within the framework of ecological justice. They propose that “in cases of ecological harm, restorative justice should be aimed at recovering those ecosystemic conditions that have been altered by the anthropic activity causing the damage, which are necessary for the affected species to flourish and develop adequately” (page no. 8) In the case of collapse of the most vulnerable and affected ecosystems, 2019), it could qualify as an ecological crime from an ecocriminological point of view. Thus, they propose from ecological restorative justice a double response involving all social actors and especially the responsible public administrations: “on the one hand, measures of prevention and mitigation; on the other hand, of repair and adaptation” (page no. 8).

 In their conclusions, following the insights of Nigel South, the authors diagnose that the activities at the source of marine pollution "are legal but generate illegitimate ecological harm to aquatic ecosystems and therefore to nature itself". This is why the authors, after their exhaustive analysis, claim the broader framework of ecological justice, to provide an ecocentric approach and new proposals to be considered in the future for the protection of *Posidonia*, the marine environment and nature. Their work is an excellent example of how green criminology can provide solutions based on a regulatory framework that needs to be revised and better implemented in order to preserve our marine ecosystems according to the imperative of ecological justice.

Is the argument persuasive and supported by appropriate evidence?

Yes

Is the work clearly and cogently presented?

Yes

Is the topic of the essay discussed accurately in the context of the current literature?

Yes

Does the essay contribute to the cultural, historical, social understanding of the field?

Yes

Reviewer Expertise:

European and International Environmental Law

I confirm that I have read this submission and believe that I have an appropriate level of expertise to confirm that it is of an acceptable scientific standard.

## References

[1] : Dubai’s Costly Water World. *the new york times* .2023;

# Reviewer report: Reviewer response for version 1

**DOI:** 10.21956/openreseurope.17668.r36659

**Published:** 2024-01-30

**Authors:** Fabio Blanco-Murillo

**Affiliations:** 1 University of Alicante, Alicante, Spain

The authors have analyzed and discussed the problems related to desalination brine discharges and their potentially detrimental effects on *Posidonia oceanica* meadows. From the basis of a weak legal framework, the authors highlight the potential threat of this activity on sensitive ecosystems such as *P. oceanica* meadows. 

 However, there are information gaps and issues that require revision .

**Introduction:**

 Although this could be an object of orthographic discussion, we generally refer to the industry as "Desalination" instead of "Desalinisation".

 Also, is more correct to express a scientific name as " *P. oceanica*" rather than " *Posidonia*".

 I also recommend adding the recently published work of Blanco-Murillo et al. (2023) to compliment Cambridge et al., (2019), highlighting the detrimental role brine might have on *P. oceanica* beyond hypersalinity itself.

 Which scientific databases were consulted?

**Approach to brine as an environmental problem from the point of view of ecocriminology**

 When explaining the osmosis process, you should mention that only water can move through semipermeable membranes, not the whole solution.

 You should mention that the post-treatment is done on the permeate from the reverse osmosis process.

 When talking about Spanish legislation and EIA you should also mention Sola et al., 2020. They analyzed the environmental requirements of desalination projects on a large spatial and temporal scale.

 I would avoid references to local news and subjective concepts such as "poison dor Posidonia". Even if we know that brine discharges are harmful to *P. oceanica *meadows, the analyses must be based on scientific and proven evidence. There are cases of sustainable desalination projects with very low affection on the *P. oceanica* meadows nearby (see Sola et al., 2019)

 Be careful with the statements. Brine can present salinities of 60-70 psu (is more common to use psu than g/l), but that does not mean those salinities are detectable in the marine environment. Natural dispersion usually reduces salinity to 40-45 in the first meters (Fernandez-Torquemada et al., 2009)

 Sodium chloride (common salt), magnesium chloride, and potassium sulfate are not chemicals used for the desalination process, they are salts naturally present in seawater. Iron chloride or metabisulfite are additives commonly used in reverse osmosis plants.

 You may also mention the role of the stress duration and possible recovery of *P. oceanica* under hypersaline stress (Marín-Guirao et al., 2013) or the effect of sodium metabisulfite on a seagrass species (Portillo et al., 2014)

 I do not agree with the comments about Malefeito et al. (2005). If it is a small area, it should not be able to assume a higher volume of water, on the contrary. The smaller the receptor water mass, the higher the detected salinities. Javea desalination plant is quite small in comparison to others and receives a dilution of 4:1 discharging a very diluted brine. Moreover, there is a *P. oceanica* meadow less than 100 meters away from the Fontana channel, I would not say that if "far" from it (see Sola et al., 2019). Please, rewrite this paragraph:

 ["This is a small area, capable of assuming a greater volume of discharge, with a better gradient, less biological diversity and far from the *Posidonia* meadows (page no. 91). The area affected by the brine, therefore, has not resulted in significant impacts on this species (pp. 93–94)."]

 [...reaching more than 90% of species of this taxon in the areas near the outfall of the discharge.]

 Which taxon do you refer to? Polychaetes?

 As well as in the Blanco-Murillo et al., 2020 study, Talamanca Bay is affected by several stressors. There is no doubt the desalination plant is one of them but, it is not possible to "ensure" that *P. oceanica *retreat is exclusively caused by brine. That should be mentioned because your statements seem to indicate all the meadow loss is caused by a single stressor. Boat anchoring and water quality loss caused by other human activities might worsen the effect of brine and all these impacts should be considered when facing restorative projects. Ecosystems should be studied as a whole but anthropogenic impacts should be too.

 The analyses of other species affected by brine discharges should be based on published bibliography. Just by assuming that seagrass-associated fauna is going to be affected if the meadow is lost, there is no new information added to the problem. Check de la Ossa-Carretero et al., 2016 as an example of influenced faune by brine discharges.

*Pinna nobilis* is the largest bivalve in the Mediterranean, there are other mollusks (cephalopods) that can be bigger in the Mediterranean.

**Proposals for restorative justice**

 Brine mining is currently being studied and no real rentable projects are working at the scale of desalination projects. Furthermore, this industry has its impacts (space for evaporation ponds, chemical treatments), so I do not think is something to be considered as a mitigation strategy.

 ["seedling from seeds"] is repetitive (seedling by definition always comes from seeds).

 I believe you could offer more exact restorative measures for restorative justice. 

 For example, you could recommend forcing the industry to invest in restorative projects that cover X% of the meadow area loss. Or applying modifications to the discharge (diffusers, preventive dilution) when salinity thresholds are exceeded.

Is the argument persuasive and supported by appropriate evidence?

Partly

Is the work clearly and cogently presented?

Partly

Is the topic of the essay discussed accurately in the context of the current literature?

Partly

Does the essay contribute to the cultural, historical, social understanding of the field?

Yes

Reviewer Expertise:

Environmental impact of desalination projects and ecotoxicology

I confirm that I have read this submission and believe that I have an appropriate level of expertise to confirm that it is of an acceptable scientific standard, however I have significant reservations, as outlined above.

## References

[1] : Desalination brine effects beyond excess salinity: Unravelling specific stress signaling and tolerance responses in the seagrass Posidonia oceanica. *Chemosphere* .2023;341: 10.1016/j.chemosphere.2023.140061 140061 10.1016/j.chemosphere.2023.140061 37689149

[2] : Sustainable desalination: Long-term monitoring of brine discharge in the marine environment. *Mar Pollut Bull* .2020;161(Pt B) : 10.1016/j.marpolbul.2020.111813 111813 10.1016/j.marpolbul.2020.111813 33157504

[3] : Review of the management of brine discharges in Spain. *Ocean & Coastal Management* .2020;196: 10.1016/j.ocecoaman.2020.105301 10.1016/j.ocecoaman.2020.105301

[4] : Dispersion of brine discharge from seawater reverse osmosis desalination plants. *Desalination and Water Treatment* .2009;5(1-3) : 10.5004/dwt.2009.576 137-145 10.5004/dwt.2009.576

[5] : Bioindicators as metrics for environmental monitoring of desalination plant discharges. *Mar Pollut Bull* .2016;103(1-2) : 10.1016/j.marpolbul.2015.12.023 313-318 10.1016/j.marpolbul.2015.12.023 26781455

# Response

**Published:** 2024-02-01

**Authors:** Esteban Morelle-Hungría

**Affiliations:** Public Law, Universitat Jaume I, Castellón de la Plana, Valencian Community, Spain

Dear reviewer, thank you for your review and input. After receiving the three revisions, most of your suggestions and comments have been incorporated into the new version of the paper. We believe that almost all of your contributions have been incorporated, including a new bibliography, which we believe has significantly improved the outcome.

# Reviewer report: Reviewer response for version 1

**DOI:** 10.21956/openreseurope.17668.r36652

**Published:** 2024-01-03

**Authors:** Gema Varona

**Affiliations:** 1 University of Basque Country, Leioa, Spain

This article addresses the ecological harm caused by desalination and brine discharges on a protected specie, *Posidonia oceanica, *in the Balearic Islands, from an ecological restorative approach.

 It is an important piece of research that contributes to the emerging field of green criminology (or eco-criminology) in Spain with a concrete topic of great interest in what has been termed as 'blue (restorative) justice' that entails the need of interdisciplinarity.

 The first reviewer has already noted some suggestions in terms of ecology. In my case, beyond some minor editing issues, I can contribute with some notes on Criminology, Sociology of Law and Restorative Justice studies.

 First, in the Introduction (at the end of the 3rd paragraph), some quotation is needed in relation to the 'systems approach' used by the authors which I tend to relate to Sociology of Law.

 In relation to the whole topic of Restorative Justice, more emphasis should be given to repairing human and non human relationships, that is, there are many more harms than ecological harms in terms of biology and ecology studies. Restorative Justice always addresses the immaterial harm suffered (in this case by ecosystems, species and individuals themselves, being human or non human). In fact, the key issue here is how there can be a restorative dialogue or a meaningful conversation entailing the different agents involved, with very diverse interests in conflict -as the first reviewer underlines-, including diverse generations, species, individuals, legal persons, authorities, etcetera. I personally miss more concrete details on the process of restorative justice in this realm that, we know, it can be very flexible and creative and, yet, it has to be transformative to avoid restorative green washing.

 I think the readers and the authors can greatly benefit from the reflection on these details, perhaps with just a draft or scheme of the concrete steps to be given mentioning those involved and how the role of Restorative Justice facilitators and the community is crucial here to build the trust needed for a form of justice co-constructed on a voluntary basis.

Is the argument persuasive and supported by appropriate evidence?

Partly

Is the work clearly and cogently presented?

Yes

Is the topic of the essay discussed accurately in the context of the current literature?

Partly

Does the essay contribute to the cultural, historical, social understanding of the field?

Yes

Reviewer Expertise:

Criminology, Victimology, Restorative Justice

I confirm that I have read this submission and believe that I have an appropriate level of expertise to confirm that it is of an acceptable scientific standard, however I have significant reservations, as outlined above.

# Response

**Published:** 2024-02-01

**Authors:** Esteban Morelle-Hungría

**Affiliations:** Public Law, Universitat Jaume I, Castellón de la Plana, Valencian Community, Spain

Dear reviewer, 

 We would like to thank you for your work in reviewing our paper. We have used your comments and suggestions to improve the first version of our research. We have included the detailed technical aspects and significantly expanded the analysis from a restorative justice perspective. We believe that almost all of your comments and contributions have been met, which has significantly improved the new version that has been sent to the editor.

 Yours sincerely,

# Reviewer report: Reviewer response for version 1

**DOI:** 10.21956/openreseurope.17668.r35054

**Published:** 2023-09-25

**Authors:** Jordi Pagès

**Affiliations:** 1 Centre d'Estudis Avançats de Blanes, Girona, Spain

The essay by Morelle-Hungría and Serra presents the interesting and (at least to me) novel development of eco-criminology and ecological restorative justice, using brine discharges on Posidonia oceanica meadows as a case study. I enjoyed reading a paper that is so far away from my field of expertise – ecology of coastal vegetated ecosystems (sand dunes, saltmarshes and seagrass meadows).

 I generally found the ecological evidence base supporting the link between brine disposal and Posidonia meadows’ degradation well substantiated. In the vocabulary used by the authors, the link between the pressure and the ecological harm is well researched and the conclusion is clear: hypersalinity produces mortality and degradation of Posidonia plants and meadows.

 However, there is a problem of scale with the argument presented in the paper. While the link between pressure and harm is obvious and scientifically established; as far as I am aware, the scale of degradation is very restricted (on the scale of tens or hundreds of metres around the outlet of the desalination plant). Therefore, the harm, in general, appears to be spatially restricted, while the benefit (access to drinking water) is large. As a result, I believe the manuscript should address the ethical dilemma posed by the need for desalination to provide access to clean drinking water, especially in arid regions where freshwater resources are scarce. The right to safe and clean drinking water is recognized as a fundamental human right by the United Nations. Discussing this aspect is critical for achieving a balanced perspective on the issue.

 Moreover, the paper discusses various salinity thresholds beyond which ecological harm is observed. However, the establishment of these thresholds can be complex and may vary depending on location and ecosystem characteristics. It's important to highlight any uncertainties or variations in the scientific consensus on these thresholds.

 It is also essential to acknowledge that environmental protection and human rights often involve trade-offs and complex policy choices. The paper should explore potential strategies for minimizing ecological harm while still meeting the critical water needs of communities. This might involve innovative brine disposal methods (e.g. diffusers to dilute the brine faster), improved technology, or stricter regulatory oversight.

 Finally, there are some inaccuracies in the use of ecological/scientific terms and in the use of citations throughout the text. For example, primary production is the process whereby inorganic matter is transformed to organic matter, usually by plants via photosynthesis. Therefore, animals, in general, cannot be primary producers as is implied in page 7 (or maybe you meant that animals consume primary production from Posidonia? Then it is correct, but not very well articulated). Also, sodium chloride is also a mineral salt (page 5), it is NaCl, sea salt. Also, as far as I am aware, the ecological restoration of P. oceanica has not been much successful, while the reverse is implied in page 8. In the same page, the citations Pauly 2019 and Schmitz et al. 2023 are not the most adequate for the context in discussion.

Is the argument persuasive and supported by appropriate evidence?

Partly

Is the work clearly and cogently presented?

Partly

Is the topic of the essay discussed accurately in the context of the current literature?

Partly

Does the essay contribute to the cultural, historical, social understanding of the field?

Yes

Reviewer Expertise:

Ecology

I confirm that I have read this submission and believe that I have an appropriate level of expertise to confirm that it is of an acceptable scientific standard, however I have significant reservations, as outlined above.

# Response

**Published:** 2024-02-01

**Authors:** Esteban Morelle-Hungría

**Affiliations:** Public Law, Universitat Jaume I, Castellón de la Plana, Valencian Community, Spain

Dear reviewer, We would like to thank you for your work in revising our article, especially for your technical assessments, which have been incorporated into the new version. We have also included, as you suggested, a brief introduction to an ethical discussion of the use of these practices, taking into account the impacts generated in the current drought situation. As indicated, most of your contributions and suggestions have been incorporated into the new version, as well as your technical comments, which have undoubtedly improved the final result significantly. Sincerely,
